# Critical resolution: A superior measure of crowding

**DOI:** 10.1016/j.visres.2018.08.005

**Published:** 2018-12

**Authors:** Leili Soo, Ramakrishna Chakravarthi, Søren K. Andersen

**Affiliations:** School of Psychology, University of Aberdeen, United Kingdom

**Keywords:** Object recognition, Visual crowding, Psychophysics, Critical spacing, Critical resolution, Visual perception, Flanker interference

## Abstract

Visual object recognition is essential for adaptive interactions with the environment. It is fundamentally limited by crowding, a breakdown of object recognition in clutter. The spatial extent over which crowding occurs is proportional to the eccentricity of the target object, but nevertheless varies substantially depending on various stimulus factors (e.g. viewing time, contrast). However, a lack of studies jointly manipulating such factors precludes predictions of crowding in more heterogeneous scenes, such as the majority of real life situations.

To establish how such co-occurring variations affect crowding, we manipulated combinations of 1) flanker contrast and backward masking, 2) flanker contrast and presentation duration, and 3) flanker preview and pop-out while measuring participants’ ability to correctly report the orientation of a target stimulus. In all three experiments, combining two manipulations consistently modulated the spatial extent of crowding in a way that could not be predicted from an additive combination. However, a simple transformation of the measurement scale completely abolished these interactions and all effects became additive. Precise quantitative predictions of the magnitude of crowding when combining multiple manipulations are thus possible when it is expressed in terms of what we label the ‘critical resolution’. Critical resolution is proportional to the inverse of the smallest flanker free area surrounding the target object necessary for its unimpaired identification. It offers a more parsimonious description of crowding than the traditionally used critical spacing and may thus constitute a measure of fundamental importance for understanding object recognition.

## 1 Introduction

Object recognition is essential for visually guided adaptive behaviour. For example, while driving on a rainy evening, timely recognition of a pedestrian about to cross the street may be essential to avoiding an accident. Our ability to recognise an object in the periphery as a pedestrian would be impaired if she were standing next to an object of similar size and shape, such as for example, a road sign. This reduction in the ability to identify objects in clutter is called visual crowding (Bouma, 1970, Levi, 2008, Pelli and Tillman, 2008, Pelli et al., 2004, Whitney and Levi, 2011). Crowding fundamentally limits our ability to process visual scenes as diverse as driving, reading or searching for a particular object. In most situations crowding, rather than visual acuity, is the limiting factor on visual perception. In recent years, substantial efforts have been undertaken to uncover the limits of object recognition, using crowding as a tool (Chung et al., 2001, Harrison and Bex, 2015, He et al., 1996, Herzog and Manassi, 2015, Herzog et al., 2016, Pelli et al., 2004).

The Bouma Law (coined by Pelli & Tillman, 2008) describes one of the most fundamental properties of crowding. It states that the distance between a target and its flankers below which the flankers start to interfere with the identification of the target is proportional to the target’s eccentricity, i.e. its distance from fixation (Bouma, 1970). This distance between target and flankers is known as the ‘critical spacing’ and is considered to be the measure that best characterises the interference between nearby objects. It was initially reported to be approximately half the target’s eccentricity (Bouma, 1970). There is evidence that the Bouma Law holds true for a large variety of objects and features, such as orientation, hue, lightness, size (van den Berg, Roerdink, & Cornelissen, 2007), spatial frequency (Chung et al., 2001), letters (Bouma, 1970, Kooi et al., 1994, Pelli et al., 2004, Wolford and Chambers, 1984), faces (Farzin, Rivera, & Whitney, 2009), real-world objects (Wallace & Tjan, 2011) and natural scenes (Wallis & Bex, 2012). This consistency has led some researchers to propose the Bouma Law as a general principle of object recognition (Pelli & Tillman, 2008) that has implications for the neural mechanisms of feature integration. According to this idea, neurons (in say V1) responding to object features will pool their responses if they are within a certain distance (6 mm in the radial direction) of each other in the cortex (Pelli, 2008), leading to crowding.

However, this notion seems inconsistent with studies that have revealed large variations in the proportionality constant that links critical spacing and eccentricity. For example, critical spacing is reduced (less target-flanker interference) if target and flankers differ in some property such as colour (Andriessen and Bouma, 1976, Chung et al., 2001, Kennedy and Whitaker, 2010, Kooi et al., 1994, Nazir, 1992, Põder, 2007, Scolari et al., 2007) or if the flankers are previewed (Scolari et al., 2007, Watson and Humphreys, 1997). On the other hand, critical spacing is increased, and indeed can be much larger than half the eccentricity, if the flankers’ luminance contrast is higher than that of the target (Rashal & Yeshurun, 2014), if the target is mildly masked, (Vickery, Shim, Chakravarthi, Jiang, & Luedeman, 2009), or if display duration is reduced (Kooi et al., 1994, Tripathy et al., 2014), whereas masking the flankers reduces critical spacing (Chakravarthi and Cavanagh, 2009, Wallis and Bex, 2011).

These findings suggest substantial variability in the distance over which features are integrated, depending on stimulus properties. Thus, the amount of crowding may differ vastly between dissimilar scenes or even objects within the same scene. To understand how crowding limits visual perception, it is, therefore, necessary to know how various stimulus manipulations affect crowding and what the combined effect of such manipulations is. The latter is especially important for two reasons. First, real-world scenes combine multiple object properties in a variety of ways. For example, a flanker might differ from the target in contrast, spatial frequency, and orientation, simultaneously. In addition, effective viewing durations might vary a lot due to movements of eyes, observers, or objects. Masking can occur when an object or its flankers are occluded by other (perhaps moving) objects. In order to move towards an understanding of the limitations of object recognition in the real world, it is therefore necessary to understand the effects of combinations of stimulus properties. Second, the magnitude of the effects of different stimulus properties on crowding can only be compared across studies if they are either independent of each other or if the way in which these effects are combined is exactly understood. For example, doubling the contrast of flankers (while keeping target contrast constant) approximately doubled the critical spacing in a previous study (Rashal & Yeshurun, 2014). Would such a surprisingly large effect also have been observed if stimuli had not been presented very briefly and with a backward mask? It could even be the case that the effect of one manipulation is contingent upon a certain combination of other factors. If this were the case, manipulating flanker contrast might only have a (detectable) effect when measured under these specific conditions. Perhaps surprisingly, previous studies have typically tested the effects of manipulating stimulus properties on crowding in isolation (e.g., Kooi et al., 1994, Rashal and Yeshurun, 2014, Scolari et al., 2007). It is therefore unknown what the combined effect of such manipulations is and whether it follows a regular pattern across different manipulations.

The present study examined how the effects of stimulus properties that affect object recognition in a cluttered scene are combined. We manipulated flanker contrast together with backward masking (Experiment 1) and display duration (Experiment 2). Additionally, we manipulated flanker preview and target-flanker similarity in a third experiment (Experiment 3). We employed full-factorial designs in order to assess both main effects and interactions of these manipulations on critical spacing. This allows us to determine whether the effects of combining two properties can be predicted from the extent of visual crowding observed when manipulating these properties separately.

## 2 Methods

### 2.1 Participants

All participants were students at the University of Aberdeen. Experiment 1 had fifteen participants (11 female; 13 right-handed; mean age = 22.2 years; age range: 18–25 years), Experiment 2 had ten participants (6 female; all right-handed; mean age = 22.6 years; age range: 19–27 years) and Experiment 3 had twelve participants (8 female; 11 right-handed; mean age = 24.1 years; age range: 20–26). In all experiments, participants had normal or corrected to normal visual acuity. Participants gave written informed consent prior to participation. They received either £5 or course credits as compensation for their participation. All experiments were approved by the University of Aberdeen Psychology Ethics Committee (Project number: PEC/3146/2014/10) and the work was carried out in accordance with the Code of Ethics of the World Medical Association (Declaration of Helsinki).

### 2.2 Experiment 1

#### 2.2.1 Materials

Stimuli were generated and presented using Matlab (The MathWorks, Natick, MA) with the Cogent Graphics toolbox (developed by John Romaya, Laboratory of Neurobiology, Wellcome Department of Imaging Neuroscience) on a 19 in. CRT monitor set to a resolution of 1024 × 768 pixels and a refresh rate of 60 Hz, viewed from a distance of 60 cm. The target was the letter ‘T’ (1.3° of visual angle) and was presented 9° from fixation in either the left or right visual field along the horizontal meridian in one of four cardinal orientations: upright, inverted, rotated 90° right or 90° left. The target letter appeared either in isolation or was surrounded by three flanking stimuli (above, below and on the outer side of the target). No flanker was presented on the inner side of the target as such a flanker would have approached or intersected fixation at large target-flanker distances. Flankers were letter ‘H’s (same size as the target stimulus), presented either upright or rotated 90°. Flankers, when present, could be at one of seven possible distances from the target measured centre to centre: 1.5°, 2°, 2.5°, 3°, 4°, 5° and 7° of visual angle. The experiment manipulated the presence of backward masking and flanker contrast. The backward mask was a rectangle of size 8.2° × 26.7°, created by tiling patches of size 0.2° × 0.2°. Each individual patch of the mask had a random grey scale luminance value sampled from a uniform distribution between 0.02 and 57.44 cd/m^2^.

The Weber Contrast of stimuli was calculated as follows:

(1) $$\mathrm{contrast}=\frac{I-I_{b}}{I_{b}}$$

where *I* is the luminance of the stimulus and *I_b_* is the luminance of the background. Targets had a luminance of 19.6 cd/m^2^ corresponding to a contrast of 0.25 against the grey background (15.7 cd/m^2^). The flankers either had the same contrast as the target or had a luminance of 39.5 cd/m^2^ corresponding to a contrast of 1.5 from the background.

#### 2.2.2 Procedure

**Fig. 1:**
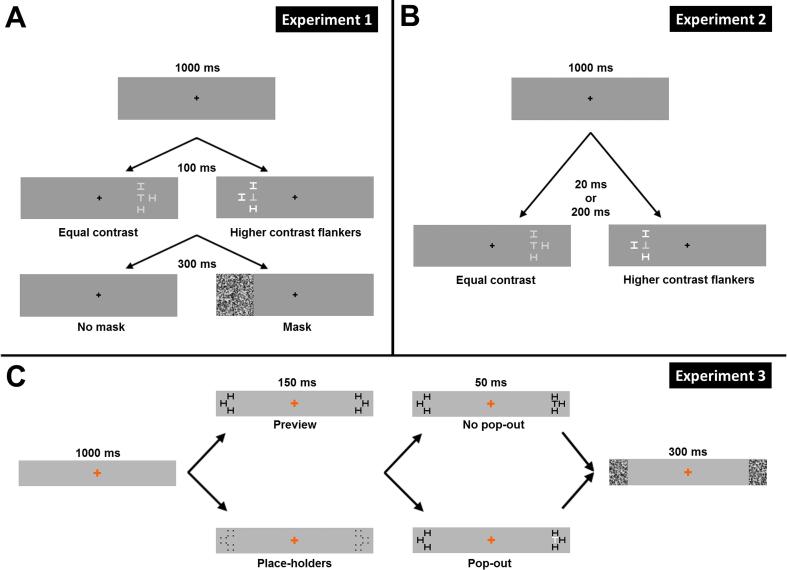
(A & B) The sequence of events in a single trial in Experiment 1 (A) and Experiment 2 (B). A fixation cross was presented in the middle of the screen throughout the experiment. The target and flankers were presented either to the right or left of the fixation (9° eccentricity). Targets (‘T’) were either presented in isolation or surrounded by equal contrast (Weber contrast of 0.25) or higher contrast (Weber contrast of 1.5) flankers (‘H’) at one of seven different target-flanker distances (closest spacing depicted in the figure). In Experiment 1, the target display was followed by a backward mask (same side as stimulus display) or no mask. In Experiment 2, target display was presented for either 20 ms or 200 ms (no masking). The next trial started immediately after participants had responded to the target orientation (up, down, left or right) by a key press. (C) The sequence of events in a single trial in Experiment 3. While participants fixated on the central cross, bilateral place-holders or flankers were presented for 150 ms at one of nine flanker distances (closest spacing depicted in the figure) and with positive or negative contrast polarity. Subsequently, a target with the same or opposite polarity was presented for 50 ms along with flankers that matched the place-holders’ contrast polarity or in isolation. The following trial started 1000 ms after participants had reported the orientation of the target.

The sequence of events during Experiment 1 are depicted in Fig. 1A. Each trial started with a fixation cross presented for 1000 ms. Then, the target and three flankers were presented for 100 ms. In half the trials, a noise mask was presented for 300 ms after the offset of the target display (target-mask SOA of 100 ms). Flanker contrast was the same as the target’s in half the trials and higher in the other half. Target and flanker orientations were randomly chosen for each trial. Participants were instructed to report the target orientation by pressing the corresponding arrow key (left, right, up or down) on a keyboard. Auditory feedback was provided on each trial; percentage correct averaged over all the trials within a block was displayed at the end of that block.

Participants underwent training for 1–3 blocks of 32 trials each, at the beginning of the experiment. The main experiment consisted of a total of 1024 trials. There were 256 different types of trials: 2 sides (L/R) × 2 flanker contrasts (equal/higher) × 2 masking conditions (yes/no) × 8 flanker distances (1.5°, 2°, 2.5°, 3°, 4°, 5°, 7° and no flankers) × 4 target orientations. Each type of trial was repeated 4 times and all trials were presented in random order. After every block of 128 trials, participants were given a self-paced break during which they received written feedback on their average accuracy in the preceding block. For purposes of analysis, data was averaged over sides and target orientations, leaving 32 conditions with 32 trials each.

#### 2.2.3 Analysis

Exponential curves were fit to the accuracy of target orientation discrimination responses as a function of target-flanker distance for each condition and each participant separately. The no flanker condition, which is virtually an infinite target-flanker distance condition, was included in these fits by assigning it the very large target-flanker distance of 20°. This particular choice of where to insert the no flanker condition on the x-axis did not affect the resulting fits noticeably as verified by re-running the analysis with other values (9–100°). The no flanker conditions are physically identical in the equal and higher flanker contrast conditions in Experiment 1 and 2, and in all conditions in Experiment 3. Accuracy was averaged across all physically identical conditions for the no-flanker condition prior to fitting (plotted separately for illustration).

The exponential function (Rashal and Yeshurun, 2014, Scolari et al., 2007, Strasburger, 2001), used to fit the data was:

(2) $$y\left(x\right)=\propto \left(1-e^{\left(-s\left(x-t\right)\right)}\right)$$

where *y* is target-identification accuracy, ∝ is the upper asymptote, *s* is the scaling factor, *x* is the target-flanker distance and *t* is the x-intercept of the curve. Lower bound for parameter ∝ was guessing chance (i.e. 0.25) and parameters t&s were restricted to be non-negative (≥0). The upper bounds were 1 (100% performance) for ∝ and 10 for *s*, which corresponds to an almost impossibly steep slope. There was no upper bound for t. The critical spacing $x_{c}$ is commonly defined as the distance, at which performance reaches 90% of the asymptote, and was computed as follows:

(3) $$x_{c}=t-log\left(0.1\right)/s$$

Critical spacing was calculated using Eq. (3) for each participant and condition separately, and the results were subjected to a 2 × 2 repeated measures ANOVA. Bonferroni-Holm correction was used for post hoc analysis. To verify that our results were not specific to the particular choice of fitting function we reran analyses with fits to cumulative Gaussian and Weibull curves, both of which yielded qualitatively identical results. In two of our experiments, the critical spacing, defined as 90% of the asymptote, was sometimes beyond the furthest stimulus spacing, i.e. we extrapolated. However, the results were qualitatively the same with a 75% of asymptote criterion, for which such extrapolation did not happen. We can, therefore, exclude the possibility that our findings were qualitatively influenced by such extrapolation.

### 2.3 Experiment 2

The design of Experiment 2 was identical to that of Experiment 1, except that we manipulated flanker contrast (equal/higher) and display duration (20 ms/200 ms). No mask was used in this experiment. Monitor refresh rate was set to 100 Hz. The sequence of events during Experiment 2 are depicted in Fig. 1B.

### 2.4 Experiment 3

The design of Experiment 3 was the same as the previous two experiments, except that here we manipulated flanker preview (preview/no preview) and target-flanker similarity (pop-out/no pop-out). We also employed backward masking, as the performance was near ceiling without it. The background was set to 24.8 cd/m^2^ light grey and the red fixation cross was isoluminant to the background. Targets and flankers were either black (luminance 14.9 cd/m^2^) or white (luminance of 34.6 cd/m^2^), both of which had a Weber contrast of ± 0.4 relative to the background. The mask was identical to Experiment 1, except that it was presented on both sides of fixation.

The sequence of events in Experiment 3 was slightly different from the previous two experiments and is depicted in Fig. 1C. After a fixation interval of 1000 ms, placeholders at the flanker locations (three on each side of fixation) were presented for 150 ms. The target was then presented for 50 ms on one side of fixation. The symmetrical location on the other side remained unoccupied. Flankers replaced the placeholders for the same duration. Flankers appeared on both sides. Immediately after the offset of the stimuli, masks were presented on both sides for 300 ms. In flanker preview conditions, flankers were presented instead of placeholders. That is, flankers were presented for 200 ms and the target was presented only during the last 50 ms of that interval. Since the preview also reduces position uncertainty of the flankers (participants will know how far the flankers will be on that trial), we presented the placeholders in the no-preview condition (Scolari et al., 2007). Hence, the only difference in the latter, relative to the preview condition, is not having previewed the flankers.

In the pop-out condition, flankers and targets had opposite contrast polarities (i.e. a black target surrounded by white flankers or a white target surrounded by black flankers) and in the no pop-out condition, flanker and target contrast polarities were the same (i.e. a black target surrounded by black flankers or a white target surrounded by white flankers). The previewed flankers and place-holders had the same contrast polarity as the subsequent flanker display. Pilot data revealed that the spatial extent of crowding was smaller than in the two previous experiments. To obtain the full range of performance as a function of target-flanker distance, a ninth flanker distance was added and the tested target-flanker distances were restricted to 1°, 1.25°, 1.5°, 2°, 2.5°, 3°, 4° and 5.5°. Target and flanker size was reduced to 0.7° visual angle. Due to including nine target flanker distances while retaining the same number of trials per condition (32 trials), the total number of trials increased to 1152. Before fitting exponential curves, accuracy in no-flanker conditions was averaged across all manipulations.

## 3 Results

### 3.1 Experiment 1: Backward masking and contrast

**Fig. 2:**
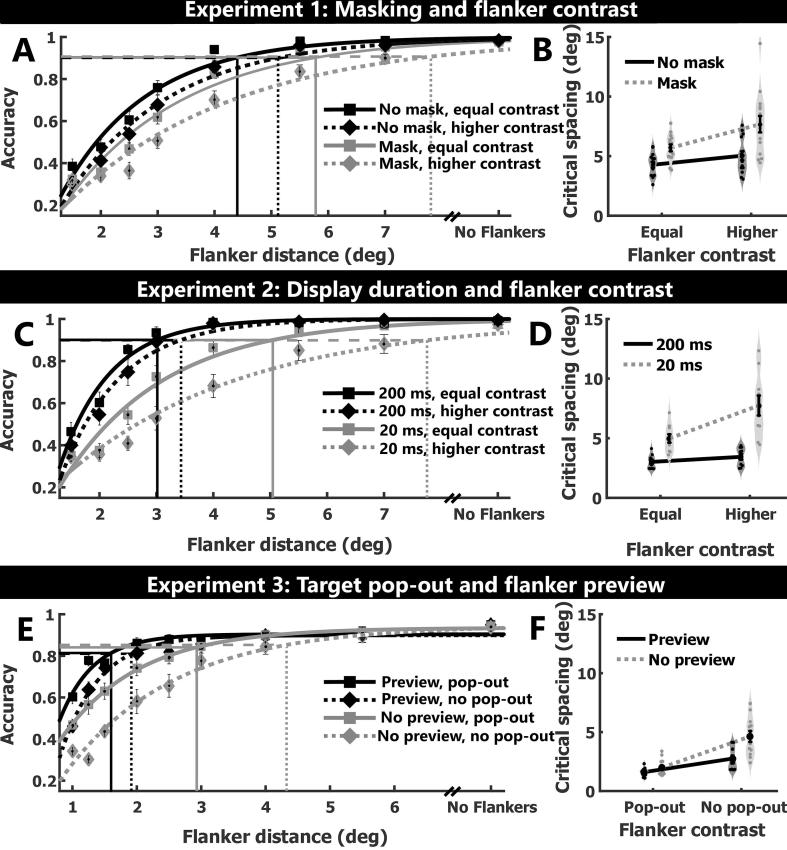
Results of experiment 1, 2 and 3. Mean accuracy as a function of the target-flanker distance (plus the No flanker condition) for each condition in experiments 1 (A), 2 (C) and 3 (E) with exponential fits for each condition. Critical spacing for each condition is computed by determining the target-flanker distance at which performance is at 90% of asymptotic performance. These are depicted by the vertical lines, drawn where the horizontal lines (90% of asymptote) intersect with the psychometric curves. (B, D, & F) Mean and standard error of the mean of the critical spacing overlaid on Violin plots (smoothened histogram with normal Kernel). Black dots depict individual participants’ critical spacing.

We independently manipulated the visibility of objects (by either presenting a subsequent mask or no mask) and flanker contrast (equal or high, relative to target contrast) while measuring the accuracy with which participants reported the orientation of a peripheral target ‘T’. We estimated critical spacing for each condition using exponential fits to accuracy performance as a function of target-flanker distance (Fig. 2A). The fit of the exponential curves to the data was excellent (mean $r^{2}$ = 0.92; range: 0.73–0.99).

The critical spacing data were subjected to a repeated measures two-way (masking x flanker contrast) ANOVA. Critical spacing (Fig. 2B) was greater when the stimuli were backward masked (6.69° ± 0.41° of visual angle) compared to when they were not masked (4.66° ± 0.23°; main effect of masking: *F*(1,14) = 39.37, *p* < 0.001, *η^2^* = 49.98%). Critical spacing was also greater when flanker contrast was higher than target contrast (6.36° ± 0.46°) compared to when they had the same contrast (4.99° ± 0.23°; main effect of flanker contrast: *F*(1,14) = 22.23, *p* < 0.001, *η^2^* = 19.61%). Importantly, critical spacing for the combination of masking and higher contrast flankers was greater than would be expected from the sum of the individual main effects (interaction between masking and flanker contrast: *F*(1,14) = 9.30, *p* = 0.009, *η^2^* = 3.91%). That is, increasing flanker contrast had a much larger effect on critical spacing when the stimuli were backward masked (post hoc pairwise comparisons between the four conditions are shown in Table 1). This becomes evident when considering the effects in relative terms: flankers with higher contrast than the target increased critical spacing by 18% without backward masking, but in the presence of masking, this effect almost doubled (34% increase). Thus, the surprisingly large effect of the contrast manipulation previously observed (Rashal & Yeshurun, 2014) was in part due to the combination with backward masking, although the effect persists to a diminished extent even in the absence of masking.

**Table 1:** Critical spacing with proportion of eccentricity in brackets (e = eccentricity = distance of the target from fixation at 9°), pairwise comparisons of critical spacing and mean change in each condition in degrees of visual angle in each experiment separately. Significant p-values (∝<0.05) indicated in bold (Bonferroni-Holm correction was used for post hoc analysis).

Experiment 1: Masking and flanker contrast					
Condition	Critical spacing	Equal contrast, no mask	High contrast flankers, no mask	Equal contrast, mask	High contrast flankers, mask

Equal contrast, no mask	4.28° (0.48 e)		+0.76°	+1.42°	+3.40°
High contrast flankers, no mask	5.04° (0.56 e)	*t*(14) = −3.74, ***p* = 0.002**		+0.66°	+2.64°
Equal contrast, mask	5.70° (0.63 e)	*t*(14) = −5.76, ***p* < 0.001**	*t*(14) = 2.43, ***p* = 0.029**		+1.98°
High contrast flankers, mask	7.68° (0.85 e)	*t*(14) = −6.16, ***p* < 0.001**	*t*(14) = −5.52, ***p* < 0.001**	*t*(14)= 4.34, ***p* < 0.001**	

Experiment 2: Display duration and flanker contrast					
Condition	Critical spacing	Equal contrast, 200 ms	Higher contrast flankers, 200 ms	Equal contrast, 20 ms	Higher contrast flankers, 20 ms

Equal contrast, 200 ms	3.01° (0.33 e)		+0.43°	+1.97°	+4.73°
Higher contrast flankers, 200 ms	3.44° (0.38 e)	*t*(9) = −3.09, ***p* = 0.014**		+1.54°	+4.30°
Equal contrast, 20 ms	4.98°0.55 e	*t*(9) = 7.72, ***p* < 0.001**	*t*(9) = 8.15, ***p* < 0.001**		+2.76°
Higher contrast flankers, 20 ms	7.74°0.86 e	*t*(9) = −6.39, ***p* < 0.001**	*t*(9) = 6.37, ***p* < 0.001**	*t*(9) = −4.88, ***p* < 0.001**	

Experiment 3: Target pop-out and flanker preview					
Condition	Critical spacing	Target pop-out, flanker preview	No pop-out, flanker preview	Target pop-out, no preview	No pop-out, no preview

Target pop-out, flanker preview	1.58° (0.18 e)		+0.39°	+1.19°	+3.15°
No pop-out, flanker preview	1.97° (0.22 e)	*t*(11) = −2.06, *p* = 0.063		+0.80°	+2.76°
Target pop-out, no preview	2.77° (0.31 e)	*t*(11) = 5.22, ***p* < 0.001**	*t*(11) = −2.86, ***p* = 0.016**		+1.96°
No pop-out, no preview	4.73° (0.53 e)	*t*(11) = 7.26, ***p* < 0.001**	*t*(11) = 5.54, ***p* < 0.001**	*t*(11) = 5.34, ***p* < 0.001**	

### 3.2 Experiment 2: Display duration and contrast

The findings of Experiment 1 show a pronounced, and larger than expected, increase in critical spacing when combining two manipulations. To test whether this super-additive interaction is specific to the particular manipulations in the first experiment (backward masking and flanker contrast) or whether it is of a more general nature, we combined the previous contrast manipulation with a manipulation of presentation duration (Fig. 1B), which is also known to affect critical spacing (Kooi et al., 1994, Tripathy et al., 2014). Experiment 2 was identical to Experiment 1, except that stimuli were presented at two different display durations (20 ms or 200 ms) without backward masking.

Critical spacing for each of the four conditions was determined as in Experiment 1. Excellent fits to the data (Fig. 2C) were obtained (mean $r^{2}$ = 0.92; range: 0.79–0.99). A repeated measures two-way (flanker contrast x display duration) ANOVA revealed that critical spacing (Fig. 2D) was larger when stimuli were presented for a shorter duration (20 ms, 6.36° ± 0.54°) than for a longer duration (200 ms, 3.23 ± 0.16; main effect of display duration: *F*(1,9) = 51.36, *p* < 0.001, *η^2^* = 57.90%). Higher contrast flankers (relative to target contrast), once again, increased critical spacing (5.59 ± 0.65) compared to equal contrast flankers (4.00° ± 0.30°; main effect of flanker contrast: *F*(1,9) = 25.32, *p* < 0.001, *η^2^* = 14.92%). The combination of short stimulus display and higher contrast flankers resulted in the highest critical spacing, which was larger than would have been predicted from the main effects (interaction: *F*(1,9) = 19.67, *p* = 0.002, *η^2^* = 8.05%). Thus, as in the first experiment, the combination of two manipulations non-additively affected critical spacing (Table 1).

In Experiment 1, we tested critical spacing at a display duration of 100 ms. The two conditions without masking in that experiment can be directly compared to the results from Experiment 2, which presented stimuli for 20 and 200 ms while manipulating flanker contrast. Although participants in the two experiments were not the same, a consistent pattern emerged: the shorter the display duration, the larger the effect of the flanker contrast manipulation. Higher contrast flankers increased critical spacing by 55% when presentation duration was 20 ms (Experiment 2), 33% at 100 ms (Experiment 1), and 14% at 200 ms. Thus, the effect of flanker contrast is substantially modulated by other factors, such as stimulus duration and visibility. This further confirms that combining manipulations of two stimulus properties leads to pronounced non-additive interactions in critical spacing.

### 3.3 Experiment 3: Flanker preview and pop-out

Experiments 1 and 2 show super-additive effects on critical spacing when two properties are combined. This raises the question of whether such an effect of property combinations might be a general rule in crowding. However, some caution is warranted before generalising these findings to other manipulations. Both experiments shared one manipulation, flanker contrast, and in both cases, the second manipulation affected the overall visibility of the stimulus display (backward masking in Experiment 1, display duration in Experiment 2). Therefore, we cannot exclude the possibility that the pattern of our results is limited to specific manipulations or combinations thereof. To test the generality of our findings we conducted a third experiment in which we chose two manipulations that both differed from the ones employed in Experiments 1 & 2, and which did not affect visibility of the entire stimulus display. It has been extensively documented that target-flanker dissimilarity (‘pop-out’) decreases the spatial extent of crowding (Andriessen and Bouma, 1976, Kooi et al., 1994, Põder, 2007) and so does previewing flankers prior to the onset of the target (Scolari et al., 2007, Watson and Humphreys, 1997). Here, we tested whether the combination of flanker preview and pop-out leads to a similar nonlinear interaction (Fig. 1C).

In this experiment, the contrast polarity of target and flankers was varied, i.e. these stimuli could either be lighter or darker than the background. Thus flankers could either have the same (no pop-out) or opposite (pop-out) contrast polarity as that of the target and be presented in advance of (preview) or simultaneously with (no preview) the target. Once again, we fitted exponential curves to the accuracy data as a function of target-flanker spacing (Fig. 2E, mean $r^{2}$ = 0.86; range: 0.51–1.00) to estimate the critical spacing in each of these conditions.

As expected, critical spacing (Fig. 2F) was reduced when flankers were previewed (1.78° ± 0.11) as compared to when only placeholders were presented in the flanker locations prior to flanker onset (3.75° ± 0.34°; main effect of flanker preview: *F*(1,11) = 39.45, *p* < 0.001, *η^2^* = 49.12%). It was also smaller when flankers had the opposite contrast polarity relative to the target (pop-out: 2.18° ± 0.19; no pop-out: 3.35° ± 0.38°; main effect of pop-out: *F*(1,11) = 40.39, *p* < 0.001, *η^2^* = 17.50%). The combination of flanker preview and pop-out further reduced the critical spacing than what each factor considered independently would predict (interaction flanker preview and pop-out: *F*(1,11) = 12.12, *p* = 0.005, *η^2^* = 7.83%), i.e. the combination of target pop-out and flanker preview non-additively affects critical spacing (post hoc pairwise comparisons between the four conditions are shown in Table 1).

## 4 Effects of combined manipulations are additive when crowding is quantified by means of ‘critical resolution’

The three experiments indicate that the combination of multiple stimulus properties generally affects critical spacing in a nonlinear super-additive manner. That is, knowing just the effects of individual manipulations of properties on critical spacing, it is not possible to predict the effect of their combination by simple addition. However, the observed interactions followed a regular pattern across all the three experiments. This suggests that there might be a general rule that explains the magnitude of the interaction between two properties as a function of the two main effects. In other words, such a general rule should allow us to predict the effect of two simultaneously varying properties on crowding given the effect of each property separately.

If such a general rule exists, then the magnitudes of each of the main effects should be correlated with the magnitude of the interaction for each experiment. This is indeed the case: participants with larger main effects also displayed larger interactions (correlations with 95% confidence intervals^1^: Experiment 1: *r_1_* = 0.65 (0.21–0.87), *r_2_* = 0.72 (0.32–0.90); Experiment 2: *r_1_* = 0.85 (0.47–0.96), *r_2_* = 0.91 (0.64–0.98); Experiment 3: *r_1_* = 0.70 (0.20–0.91), *r_2_* = 0.59 (0.03–0.87)). These strong correlations between both main effects and the interaction in all three experiments suggest that, indeed, the interaction might directly be a function of the main effects.

**Fig. 3:**
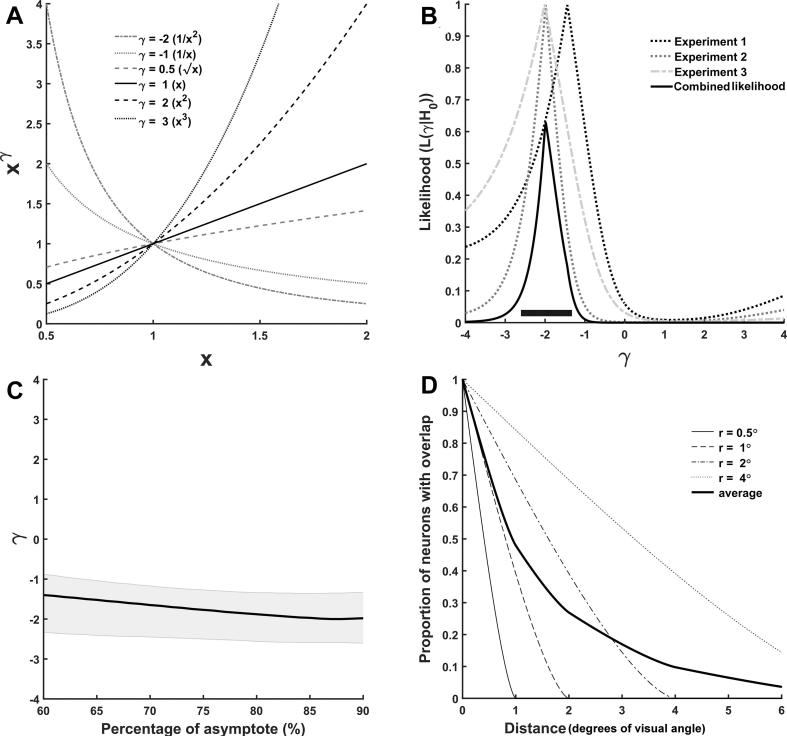
(A) Examples of power functions for different values of parameter γ. The functions are monotonically increasing for γ > 0 and monotonically decreasing for γ < 0. (B) Likelihood of different values of the exponent γ under the null hypothesis of no interaction. The combined likelihood was obtained by multiplying the three likelihoods of the separate experiments. The black bar at the bottom indicates the 14.7% likelihood region for γ. (C) The combined maximum likelihood γ as a function of the criterion (percentage of the asymptote) used to calculate the critical spacing. (D) Estimated proportion of all neurons of receptive field size r processing a target stimulus which are subject to biased competition by a flanker stimulus at distance d (see discussion for details). The displayed receptive field sizes may roughly correspond to neurons in V1 (0.5° and 1.0°), V2 (2.0°) and V4 (4.0°) (Kastner et al., 2001). Although the individual functions for neurons of the same receptive field size are almost linear for d < 2r, the function resulting from averaging over neurons of different receptive field sizes is strongly convex. Thus, a change in distance affects the extent of biased competition much more at smaller distances than at larger distances.

The attempt to discover the quantitative relationship between the main effects and the interaction in our data can be formalised as the search for a transformation $F\left(x_{c}\right)$ of the critical spacing data $x_{c}$ that minimises (or entirely removes) the interaction. Thus we ask the question what transformation of the critical spacing data would explain all the data in terms of additive main effects with no interaction effects. Towards this end, we considered the family of power functions $F\left(x_{c}\right)=x_{c}^{\gamma }$ (see Fig. 3A). Relationships in which one variable is proportional to some power of another variable are very common in many fields of technology and science. Additionally, power functions yield monotonic transformations of the data which are commonly utilised to enhance symmetry and normality of the data for statistical purposes (Box & Cox, 1964). Variation of a single parameter, γ, yields a wide variety of shapes. For γ=1, the power function is simply the identity, thus the untransformed data is explicitly included in the search space.

In order to determine an exponent γ which meets the condition of minimising the interaction, we computed the transformed critical spacing data $x_{c}^{\gamma }$ for all values of γ in the interval from −4.0 to +4.0 in steps of 0.01 for each participant (Fig. 3B). We computed the interaction term from $x_{c}^{\gamma }$ for the four conditions of each experiment and subjected the results to a one-sample *t*-test against zero to obtain the p-values of the null-hypothesis $H_{0}$ of no interaction across all participants for each experiment separately^2^. The resulting functions $p_{e}\left(H_{0}|\gamma \right)$ for each experiment e reflect the significance of the interaction after transformation of the critical spacing data (Fig. 3B) and can be interpreted as the likelihood $L_{e}\left(\gamma |H_{0}\right)$ of γ given the Null-hypothesis $H_{0}$ of the interaction. Accordingly, the maximum likelihood estimates ofγ are indicated by the peaks of these functions, which were located at −1.44, −1.98, and −2.01 for experiments 1, 2, and 3, respectively (Fig. 3B). All three experiments converged on similar values for γ, indicating that the same transformation of the critical spacing data might abolish the interaction in all three experiments and thus allow to explain all data in terms of main effects of the transformed data. The combined likelihood $L\left(\gamma |H_{0}\right)$ of the parameter γ given $H_{0}$ across all three experiments is given by the product of the three probability functions $p_{e}\left(H_{0}|\gamma \right)$:

(4) $$L\left(\gamma |H_{0}\right)=\prod_{e=1}^{3}p_{e}\left(H_{0}|\gamma \right)$$

This yields a maximum likelihood estimate of γ=-1.98 with a 14.7% likelihood region (confidence interval) from −2.60 to −1.33 (Fig. 3B). This is remarkably close to γ=-2.0. This particular estimate of γ was obtained for the definition of the critical spacing as 90% of the asymptotic performance (Eq. (3). As the 90% criterion is arbitrary, we computed the maximum likelihood γ for a range of criteria (60% of asymptote to 90%) (Fig. 3C). The parameter γ exhibited little dependency on the particular percentage of the asymptote used to calculate the critical spacing. Importantly, for all tested values the confidence interval for γ included −2.0.

For γ=-2.0, the transformation has a straightforward interpretation: the squared critical distance is proportional to the area around the target which has to be flanker-free for there to be no crowding^3^. One divided by this area is thus proportional to the highest density of objects beyond which crowding occurs under the given circumstances. We can therefore define the *critical resolution* as follows:

(5) $$\rho_{c}=1/x_{c}^{2}$$

**Fig. 4:**
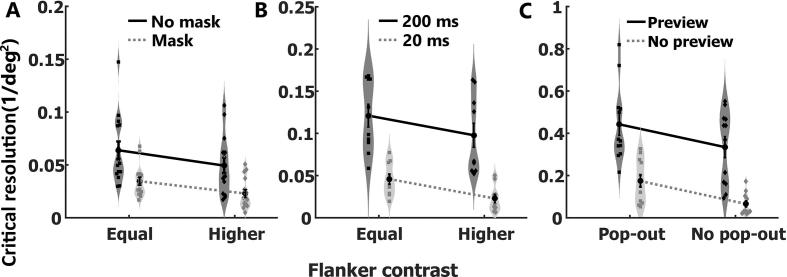
Critical resolution in Experiment 1, 2 and 3. Mean and standard error of the mean critical resolution for each of the four conditions are overlaid on Violin plots (smoothened histogram with normal Kernel). Black dots depict individual participants’ critical resolution. (A) Experiment 1, (B) Experiment 2 and (C) Experiment 3.

Expressed in terms of this critical resolution, as opposed to the critical spacing, all effects in our three experiments become independently additive (Fig. 4), such that the combined effect of varying different stimulus properties is simply the sum of their individual effects (see Table 2 and Fig. 4A–C).

**Table 2:** Critical resolution (one divided by the squared critical spacing) ANOVA results. Significant p-values (∝<0.05) indicated in bold.

Critical resolution ANOVA results			
Experiment 1	Masking: F(1,14) = 33.12, **p < 0.001**, η^2^ = 52.84%	Flanker contrast: F(1,14) = 34.88, **p < 0.001**, η^2^ = 11.65%	Interaction: F(1,14) = 0.24, p = 0.63, η^2^ = 0.14%

Experiment 2	Display duration: F(1,9) = 61.72, **p < 0.001**, η^2^ = 78.60%	Flanker contrast: F(1,9) = 69.12, **p < 0.001**, η^2^ = 7.46%	Interaction: F(1,9) = 0.97, p = 0.97, η^2^ < 0.001%

Experiment 3	Preview: F(1,11) = 73.40, **p < 0.001**, η^2^ = 59.97%	Pop-out: F(1,11) = 9.28, **p = 0.01**, η^2^ = 9.70%	Interaction: F(1,11) < 0.001, p = 0.99, η^2^ < 0.001%

This analysis can also be extended to the Bouma Law. The law states that the critical spacing $x_{c}$ is proportional to the eccentricity e:

(6) $$x_{c}=be$$

The proportionality constant b depends on a variety of factors and generally ranges between 0 and 1. Applying our definition of the critical resolution (Eq. (2), we can write the Bouma Law using the critical resolution $\rho_{c}$ rather than the critical spacing as follows:

(7) $$\rho_{c}=1/\left(b^{2}e^{2}\right)=c/e^{2}$$

The constant c is given by

(8) $$c=1/b^{2}$$

## 5 Discussion

We investigated the effect of combined manipulations of stimulus properties on object recognition in the visual periphery and obtained highly consistent results across three experiments: manipulation of flanker contrast and masking (Experiment 1), flanker contrast and display duration (Experiment 2) and pop-out and flanker preview (Experiment 3) all led to super-additive interactions in critical spacing, i.e. when combining two properties, the critical spacing was not predicted by the sum of the individual main effects. This has two important consequences: first, the spatial extent of visual crowding can vary vastly between situations in which multiple stimulus properties differ. When favourable properties are combined, crowding might be minimal or practically non-existent, whereas the combination of multiple unfavourable properties can lead to crowding across very large distances. Second, predicting the critical spacing across scenes in which parameters vary heterogeneously is difficult because the magnitude of the effect of any manipulation depends on all other stimulus properties that it is combined with. More precisely, any observed effect of a given property (say, flanker contrast) is valid for the specific set of other stimulus parameters tested in that experiment, such as stimulus duration. Changing those parameters might strongly change the magnitude of the observed effect. However, we found that when crowding was measured as the critical resolution (one divided by the squared critical spacing) the effects of qualitatively very different manipulations were combined additively, i.e. without interaction. This finding is remarkable as it allows prediction of the extent of crowding under heterogeneous viewing conditions provided that the effects of individual manipulations are known. It also allows for better comparability of the magnitude of effects obtained under different conditions, because the magnitude of any manipulation becomes independent of other manipulations when quantified by the critical resolution. This may be of particular value when comparing critical spacing effects across dissimilar experiments in the literature. We obtained qualitatively identical results when rerunning our analyses with fits to cumulative Gaussian and Weibull curves, thus our conclusions seem to be independent of the particular analytical approach used to determine the critical spacing.

We propose that measuring crowding in terms of critical resolution is advantageous relative to the traditionally used critical spacing because it allows for a straightforward prediction of the effects of multiple varying stimulus properties. Although we here obtained the critical resolution directly by transformation of the critical spacing, these two measures are conceptually different. Critical spacing is the target-flanker distance beyond which flankers do not interfere with target identification. On the other hand, critical resolution is proportional to the inverse of the smallest area of the visual field surrounding a target stimulus that needs to be flanker free for the brain to resolve this target without interference. It is thus a measure of the brain’s capacity to extract information from a given area of the visual field or retina and, like critical spacing, is a function of eccentricity and stimulus properties. For any given area of the visual field, a specific number of neurons’ receptive fields will intersect that area. Thus, critical resolution is inversely related to the amount of cortical ‘real estate’ necessary to extract information without interference. Considering the conceptual differences between critical spacing and critical resolution, it might be possible to derive direct measurement techniques of the critical resolution without recourse to critical spacing in the future.

We observed very similar interactions between combinations of dissimilar manipulations and the same transformation of the measurement scale abolished all of these interactions. The most parsimonious explanation for these results is that the interactions observed in critical spacing are largely or entirely due to non-linear properties of critical spacing as a measurement scale. The underlying principle of all three experiments was to manipulate properties that increase or decrease the strength of crowding and measure how much the spacing between targets and flankers must be changed to compensate for these effects. The interactions in our data (Fig. 2) are such that the larger the spacing needed for unimpaired target identification under a given set of conditions already is, the more the spacing needs to be further increased to compensate for a further manipulation that increases crowding. In other words: the larger the spacing, the less effective any additional increase in spacing.

In the following, we will derive a hypothetical explanation for this pattern based on principles of biased competition models (Bundesen, 1990, Desimone and Duncan, 1995, Reynolds and Heeger, 2009). The central idea of such models is that stimuli compete for neuronal representation when multiple stimuli fall into the receptive field of the same neuron (Moran & Desimone, 1985). This approach has previously been used to derive a quantitative explanation of crowding data (Kyllingsbaek, Valla, Vanrie, & Bundesen, 2007) based on the idea that crowding results from such competitive interactions between stimuli. The extent of competition for processing resources depends on how many neurons have both stimuli within their receptive fields. An estimate of the proportion of such neurons as a function of the spatial separation between stimuli can be derived as follows:

The centres of the receptive fields of all neurons that process a given stimulus lie within a circle of a radius equal to their receptive field size r around that stimulus. The area of a circle of radius r is given by

(9) $$A=\pi r^{2}$$

If we assume that neurons are distributed fairly homogenously within the part of the visual field of interest, the number of neurons with receptive field size r processing this stimulus will be proportional to this area. If we now consider two stimuli placed at a distance d, then the receptive field centres of all neurons with both stimuli within their receptive fields lie within the intersection of two circles of equal radius r whose centres are separated by d. This area is given by Eq. (10):

(10) $$A=2r^{2}\cos^{-1}\left(\frac{d}{2r}\right)-\frac{d}{2}\sqrt{4r^{2}-d^{2}}$$

Thus we can estimate the proportion p of all neurons of receptive field size r that process a target stimulus and which also have a flanker stimulus at distance d within their receptive fields by dividing Eq. (10) by Eq. (9):

(11) $$p\left(d\right)=\frac{2}{\pi }\cos^{-1}\left(\frac{d}{2r}\right)-\frac{d}{2\pi r^{2}}\sqrt{4r^{2}-d^{2}}$$

The function $p\left(d\right)$ estimates the fraction of all neurons of receptive field size r processing a target stimulus which are subject to biased competition by a flanker stimulus at distance d (Fig. 3D). Thus $p\left(d\right)$ is an estimate of the competition for processing resources between two stimuli. If one considers only neurons of one specific receptive field size r, then the competition for neuronal processing between the two stimuli decreases fairly linearly as the separation d between the stimuli increases, until it reaches zero for d>2r. If, however, we consider a mixture of neurons with very different receptive field sizes (‘average’ in Fig. 3D), then further increasing the distance between objects reduces competition drastically at small spacings but only has very little effect at larger spacings. These simple^4^ geometric ideas thus yield an explanation for non-linear effects of changes in object spacing consistent with the pattern of interactions observed in our data. From this perspective, the transformation to critical resolution with γ=-2.0 (Fig. 3A) compensates for non-linear effects of changes in object spacing, such as those derived here (Fig. 3D) and potentially others related to information integration across neurons and decision making. Therefore, independent manipulations yield independent (additive) effects when measured in terms of critical resolution, but not critical spacing. In agreement with our ideas above, the biased competition model (Desimone & Duncan, 1995) assumes that competition for neuronal representation occurs at many levels of the visual processing system and thus involves neurons with very different receptive field sizes. This is also consistent with the large variability of critical spacing across conditions observed in our data.

There is considerable debate regarding the locus of crowding in the visual system (e.g., Levi, 2008). Findings from recent imaging studies disagree, but generally point to crowding occurring at multiple stages of visual processing (Anderson et al., 2012, Freeman et al., 2011, Kwon et al., 2014; also see Chen et al., 2014). Similarly, several behavioural experiments have argued for interference at different stages of the visual hierarchy (Blake et al., 2006, Chakravarthi and Cavanagh, 2009, Dakin et al., 2011, Farzin et al., 2009, Louie et al., 2007, Wallis and Bex, 2011). These lend credence to our hypothetical explanation that neurons with receptive fields of different sizes contribute towards target-flanker interactions, which can in turn explain the interactions found in our study.

Interestingly, it has been posited that objects must be separated by a certain distance on the cortical surface (6 mm in the radial direction and 1 mm in the tangential direction in V1) to be resolved without interference (Motter and Simoni, 2007, Pelli, 2008). That is, objects must be cortically separated to avoid crowding. This has been interpreted to suggest that pooling occurs over a fixed set of neurons and if more than one object activates these neurons, their features are pooled, leading to crowding. Note that this conceptualisation of pooling does not require pooling to occur in V1, but can occur in any one (or more) of the retinotopic areas. Our proposal modifies this hypothesis by suggesting that the ability to resolve an object is inversely related to the cortical *area* necessary to extract information without interference. One crucial difference with the former proposal is that we do not suggest that there is a fixed number of neurons that pool information. The critical resolution, and hence the cortical area required for identification, varies according to stimulus properties (duration, masking, contrast, etc.). A larger area is needed to resolve an object under some circumstances, compared to others. This variability might be a consequence of varying attentional recruitment of neurons (Chen et al., 2014, He et al., 1996) or simply competition for resources between objects (Scalf & Beck, 2010) under different circumstances. For example, an object presented with low contrast or for a short duration might need the recruitment of a larger number of neurons to process it with a high signal-to-noise ratio. Hence such objects need a larger flanker-free area to avoid crowding, whereas at higher contrast or longer duration a smaller area would suffice for appropriate behavioural performance. Similarly, attention (or grouping mechanisms) might aid segmentation of targets that are dissimilar to the flankers or when presented among previewed flankers, and hence reduce the number of neurons necessary for processing their identity. Whatever the mechanism that renders critical resolution sensitive to stimulus properties, our findings suggest that this resolution is additively (independently) affected when multiple stimulus properties are manipulated.

A key observation in our experiments is that the magnitude of the effect of one manipulation on object recognition is dependent on other stimulus parameters when expressed in terms of the critical spacing. This helps understand some previous findings, such as for example, the very large effect of a contrast manipulation on critical spacing in one study (Rashal & Yeshurun, 2014). This study employed both backward masking and very short display durations, both of which should have increased the effect of the contrast manipulation on critical spacing. The opposite pattern emerges when multiple favourable stimulus properties are combined. In this case, the effect of any manipulation is reduced which may make it harder to detect reliably. For example, in Experiment 3 the well-known effect of pop-out on critical spacing (Põder, 2007, Scolari et al., 2007) was only marginally significant when comparing the two conditions with preview (Table 1). Taken out of context, one could have concluded that the effect of pop-out is abolished when combined with preview. The incorrectness of this conclusion becomes easily apparent when the same data is expressed in terms of the critical resolution (Table 2, Fig. 4C). As can be seen from these examples, quantifying crowding in terms of critical resolution instead of the critical spacing enhances comparability across conditions and experiments because the magnitude of effects becomes independent of other manipulations.

Critical resolution, as a tool, is agnostic about the underlying mechanism of crowding. We argue that it is simply a better measure of crowding. Our ideas were presented in the context of the biased competition model above, but the utility of critical resolution is independent of whether one adopts this particular theoretical explanation. The idea of a limited resolution is similar to the attentional hypothesis of crowding (He et al., 1996), which posits that crowding arises when the resolution of selective attention is insufficient to focus on the target stimulus. However, it is also compatible with bottom-up models of crowding such as pooling, averaging (Greenwood et al., 2009, Parkes et al., 2001), and flanker substitution (Ester et al., 2014, Nandy and Tjan, 2007).

Some recent studies have determined that the effects of grouping on crowding challenge long-standing conclusions about crowding, such as the Bouma Law; these findings might also question the general validity of critical resolution as a measure of crowding. For example, it has been shown that flankers presented at distances far exceeding half the target eccentricity can alleviate crowding if they group with each other (Herzog, Sayim, Chicherov, & Manassi, 2015, but see Van der Burg, Olivers, & Cass, 2017). In other words, manipulating objects outside the critical spacing modulates crowding. On the face of it, this conclusion appears to contradict the notion underlying critical spacing and thus critical resolution. However, one way to reconcile these opposing findings is to consider that grouping might occur before the resolution bottleneck comes into play. That is, segmentation of feature sets occurs first, via grouping. This segmentation renders the neurons that represent these grouped feature sets functionally non-overlapping, allowing them to escape mutual interference. Hence the critical resolution for identifying the target will be high. According to this explanation, the pop-out manipulation in our third experiment reduced crowding by segmenting the target and flankers into separate feature sets.

We found a highly consistent pattern of additive effects on critical resolution across three experiments testing different combinations of flanker contrast, backward masking, display duration, pop-out and preview, all of which were previously known to affect the critical spacing (Andriessen and Bouma, 1976, Chakravarthi and Cavanagh, 2009, Chung et al., 2001, Kennedy and Whitaker, 2010, Kooi et al., 1994, Nazir, 1992, Põder, 2007, Rashal and Yeshurun, 2014, Scolari et al., 2007, Vickery et al., 2009, Wallis and Bex, 2012, Watson and Humphreys, 1997). However, we cannot exclude the possibility that the regularity we observed here does not extend to any combination of properties that affect the critical distance. Although we tested a variety of manipulations, other manipulations can also affect the critical distance, for example, attention (Põder, 2006, Strasburger, 2007, Yeshurun and Rashal, 2010). Attention is conceptually distinct from the other manipulations as it affects an internal variable rather than the stimulus display. It remains for future work to assess whether our pattern of results holds up for all of these factors and their combinations.

## 6 Conclusion

Manipulating different properties of stimuli in peripheral vision leads to non-additive interactions on the spatial extent of crowding (critical spacing). These interactions are quantitatively similar across different combinations of manipulations and become additive when crowding is quantified in terms of critical resolution. We propose that the critical resolution is a superior measure of crowding which facilitates understanding the limits of visual object recognition in the visual periphery across heterogeneous scenes.

## 7 Author contributions

Conceptualization, L.S., R.C. and S.K.A.; Methodology, L.S., R.C. and S.K.A.; Software, L.S. and S.K.A; Formal Analysis, L.S. and S.K.A.; Investigation, L.S.; Writing – Original Draft, L.S.; Writing – Review & Editing, L.S., R.C. and S.K.A.; Visualization, L.S.; Funding Acquisition, L.S., R.C. and S.K.A.; Supervision, R.C. and S.K.A.

## 8 Funding

This work was supported by the Biotechnology and Biological Sciences Research Council (BBSRC) [grant number BB/J01446X/1]. We would like to thank our anonymous reviewers for their thoughtful feedback, which helped us fine-tune our proposal about critical resolution.

## 9 Declaration of interest

None.

## Footnotes

1 Computed using the ‘corrcoef’ function in Matlab.

2 The interaction of a factorial 2x2 ANOVA is equivalent to a *t*-test of $x_{11}+x_{22}-x_{12}-x_{21}$ against zero, where x denotes the dependent variable and the indices denote the levels of the two factors. A significant interaction thus indicates a departure from an additive combination of the two main effects.

3 The squared critical distance is only proportional, but not equal, to the area: if e.g. we assume a circular shape, then the actual area of that circle would be obtained by further multiplying by π.

4 The presented derivation is for illustration of underlying principles. Many aspects are highly simplified, e.g. we ignore anisotropies of the distribution of neurons across the visual field, the preponderance of neurons with different receptive field sizes and their functional specialisations, etc.
